# Medical Legislation—Protection

**Published:** 1869-01-15

**Authors:** 


### EDITORIAL.

### *Medical Legislation.—Protection.*

If the assembled wisdom of the State Legislature pass any bills for the regulation of the practice of medicine and surgery in this Commonwealth, we trust they will not assume that it is in any sense for the protection or advantage of the scientific medical profession. Educated medical men do not ask for protection—they need no bolstering up, or the application of legislative splints or bandages. If such a bill as is contemplated becomes a law, we shall expect it to be considered as testimony that the people of the State can not take care of themselves. Accordingly to all precedent, such legislation has been considered a matter of persecution and intolerance by the classes at whom its penalties are aimed, and the people will not be slow to manifest their sympathies with the poor persecuted objects. We are sure that this will be the result. The question involved will enter into every bar-room caucus and every swindling political convention. The severer the penalty, the more certain and profound the reaction.

There is not in history an instance of more sublime self-abnegation than is now presented in the spectacle of a great, learned and powerful profession voluntarily tendering its control to a single political officer. Can there be a more illustrious exhibition of the magnanimity which characterizes the Medical Profession?

We have no doubt his present Excellency of Illinois will discharge the duty of supreme arbiter of medical matters in this State excellently well, and we will not permit ourselves to tear away the veil which as yet conceals the name and attributes of his illustrious successor.

It is humbly hoped that if a king is given to the Æsculapian realm, he will prove neither King Log nor King Stork—but meanwhile: WHO SHALL GUARD THE SHEPHERD?

